# Lynch Syndrome and MSI-H Cancers: From Mechanisms to “Off-The-Shelf” Cancer Vaccines

**DOI:** 10.3389/fimmu.2021.757804

**Published:** 2021-09-24

**Authors:** Vladimir Roudko, Cansu Cimen Bozkus, Benjamin Greenbaum, Aimee Lucas, Robert Samstein, Nina Bhardwaj

**Affiliations:** ^1^ Department of Oncological Sciences, Icahn School of Medicine at Mount Sinai, New York, NY, United States; ^2^ Precision Immunology Institute, Icahn School of Medicine at Mount Sinai, New York, NY, United States; ^3^ Tisch Cancer Institute, Icahn School of Medicine at Mount Sinai, New York, NY, United States; ^4^ Division of Hematology and Medical Oncology, Icahn School of Medicine at Mount Sinai, New York, NY, United States; ^5^ Epidemiology and Biostatistics, Computational Oncology program, Memorial Sloan Kettering Cancer Center, New York, NY, United States; ^6^ Physiology, Biophysics & Systems Biology, Weill Cornell Medical College, New York, NY, United States; ^7^ Henry D. Janowitz Division of Gastroenterology, Samuel D. Bronfman Department of Medicine, Icahn School of Medicine at Mount Sinai, New York, NY, United States; ^8^ Department of Radiation Oncology, Mount Sinai Hospital, New York, NY, United States

**Keywords:** Lynch syndrome, MSI-H, dMMR, immunotherapy, cancer vaccine

## Abstract

Defective DNA mismatch repair (dMMR) is associated with many cancer types including colon, gastric, endometrial, ovarian, hepatobiliary tract, urinary tract, brain and skin cancers. Lynch syndrome – a hereditary cause of dMMR – confers increased lifetime risk of malignancy in different organs and tissues. These Lynch syndrome pathogenic alleles are widely present in humans at a 1:320 population frequency of a single allele and associated with an up to 80% risk of developing microsatellite unstable cancer (microsatellite instability – high, or MSI-H). Advanced MSI-H tumors can be effectively treated with checkpoint inhibitors (CPI), however, that has led to response rates of only 30-60% despite their high tumor mutational burden and favorable immune gene signatures in the tumor microenvironment (TME). We and others have characterized a subset of MSI-H associated highly recurrent frameshift mutations that yield shared immunogenic neoantigens. These frameshifts might serve as targets for off-the-shelf cancer vaccine designs. In this review we discuss the current state of research around MSI-H cancer vaccine development, its application to MSI-H and Lynch syndrome cancer patients and the utility of MSI-H as a biomarker for CPI therapy. We also summarize the tumor intrinsic mechanisms underlying the high occurrence rates of certain frameshifts in MSI-H. Finally, we provide an overview of pivotal clinical trials investigating MSI-H as a biomarker for CPI therapy and MSI-H vaccines. Overall, this review aims to inform the development of novel research paradigms and therapeutics.

## Introduction

In the United States, an individual’s lifetime risk for developing cancer is estimated to be as high as 40% (www.cancer.org, “Lifetime Risk of Developing or Dying From Cancer”). Cancer is a genetic disorder in which somatic mutations in specific genes confer a selective growth advantage for tumors. Such mutations can be inherited through the germline, which results in a hereditary predisposition to an early-onset cancer, or can occur sporadically in non-germline carriers. One example is the hereditary nonpolyposis colorectal cancer syndrome known as Lynch syndrome. Defining the genetic causes for Lynch syndrome has uncovered a connection between the cellular machinery that regulates DNA repair, cancer formation and informed clinical approaches to manage the disease.

**Figure 1:**
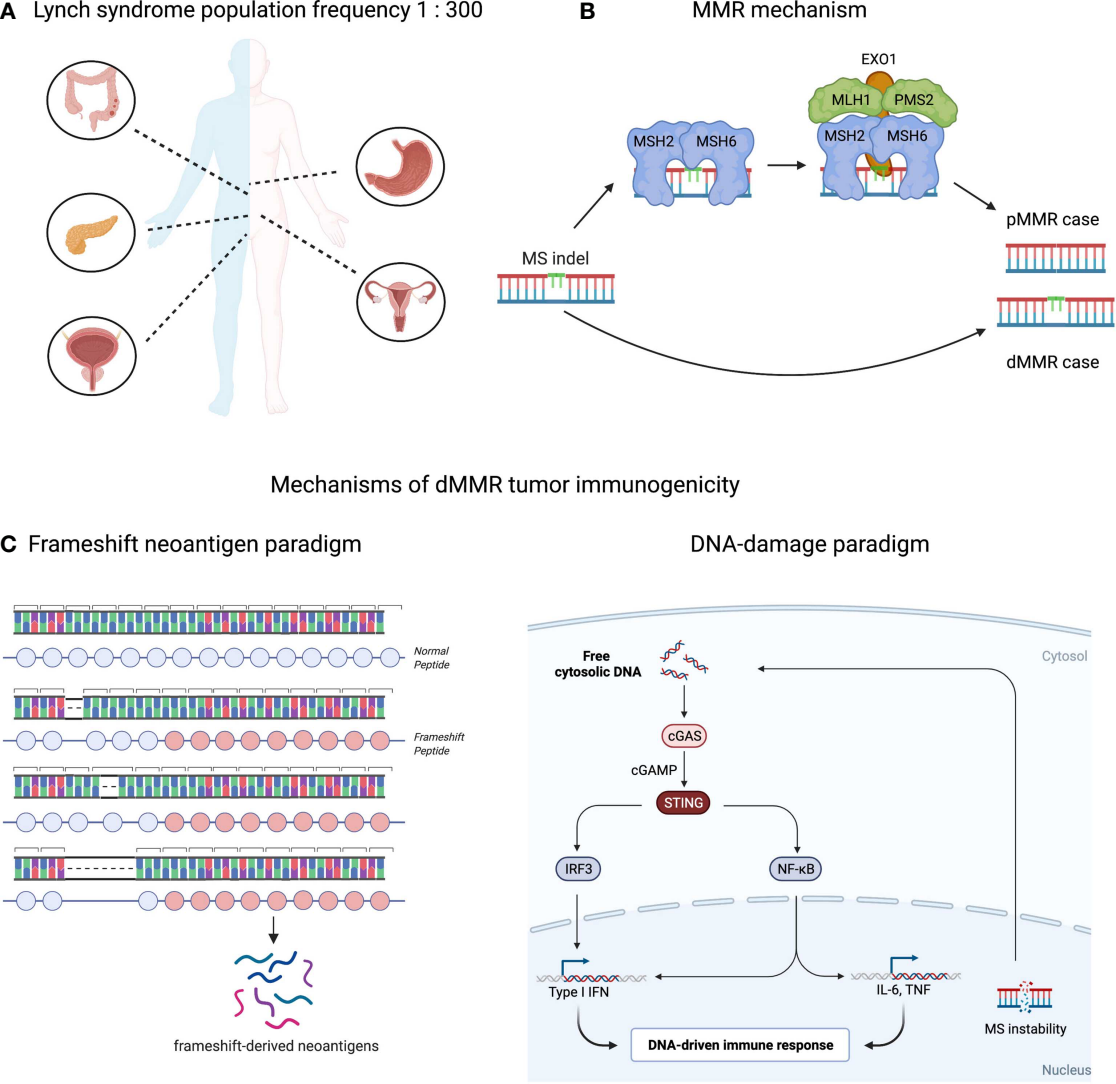
Population, molecular and immunological aspects of mismatch-repair deficient (dMMR) tumors. **(A)** Predispositions to different cancers conferred by Lynch syndrome condition. Approximately 1 in 300 people in the U.S. has the Lynch Syndrome associated alleles. Carriers have 80% lifetime risk developing cancer including: colorectal, stomach, pancreas, urinary track and prostate for males and urinary, ovary or uterus tracks for females. In total, Lynch syndrome accounts for 2-3% solid tumor cases (11). **(B)** Mismatch repair (MMR) mechanism. MS indels occurring during DNA replication are repaired by MMR system (proficient MMR). *MSH2*-*MSH3* or *MSH2*-*MSH6* complexes, called MutSα or MutSβ, detect the error and recruit the *MLH1*-*PMS1*, *MLH1*-*PMS2* or *MLH1*-*MLH3* (MutLα/β/γ complexes respectively) to bind to the DNA and bring DNA exonuclease with PCNA to the mutation site. The mismatch is then excised and repaired following by DNA resynthesis and re-ligation. These aberrations are left unrepaired in case of MMR deficiency. **(C)** Two complementary paradigms explaining immune responses in dMMR tumors: neoantigen-driven (left part), and innate immune driven (right part).

Lynch syndrome or hereditary non-polyposis colorectal cancer (HNPCC) is characterized by germline inactivation of one allele of genes involved in the mismatch repair system, namely *MLH1*, *MSH2*, *MSH6*, *PMS2 or EPCAM* and have received prominent clinical and research attention and since 1985 (1, 2). More than 1500 variants of Lynch syndrome alleles have been identified including: retrotransposition and Alu-like element insertion events (3–5), splice site mutations and large exonic deletions (6–8). However, inactivation of *MLH1* or *PMS2* alleles are the most frequent ones and are associated with approximately 80% of Lynch syndrome cases (9, 10). Lynch-associated genetic abnormalities frequently lead to cancer at ages of 30–40-years and in a broad range of tissues (**Figure 1A**), including: colon, stomach, brain, pancreas, small intestine, endometrial and urothelial tracts (12–18).

Colorectal, stomach and endometrial cancers cumulatively account for the second most common cancer types and one of the leading causes of cancer deaths in developed countries (19). The majority of the solid tumor cases are proficient in mismatch repair (pMMR), however an estimated 14% (95% CI: 10%-19%) of cases are dMMR (20–23). These dMMR cases arise mainly from sporadic, tumor-specific inactivation of MMR pathway (24, 25) however a few cases – 2-3% of all colorectal and endometrial cancer cases (11) – have germline Lynch syndrome alleles as the ones described above (26). In the latter case, cancer onset transpires upon genetic inactivation of the second allele (in the case of the *PMS2* allele) or epigenetic silencing of gene expression (in the case of the *MLH1* allele) and subsequent acquisition of driver mutations in genes such as *APC*, *KRAS*, *PI3K*, *PTEN*, *BRAF* and/or *p53* (13, 27–32). Though pMMR and dMMR share similar profiles of tumor drivers, their genomic makeups are different. One of the molecular feature of dMMR tumors is high tumor mutation burden over-represented by somatic indel mutations within short tandem repeats – microsatellites – a molecular signature termed as a high microsatellite instability, or MSI-H (27, 33–35).

## MMR Molecular Mechanism and Biomarker Strategies

Microsatellite extension or shortening in MSI-H tumors happens upon breaking down of the MMR molecular mechanism that controls microsatellite loci length (18, 36). During replication DNA polymerases incorporate deoxyribonucleotides into the growing chain of DNA using one of the paternal DNA strands as a template, thus copying genetic information with high fidelity. However, at microsatellite repeats DNA polymerases can slip from the template upon replication resulting in insertion or deletion of microsatellite units that structurally resembles a “bulge” of non-complemented DNA nucleotides within the parent/daughter DNA double strand helix. The MMR system guards against this type of mutagenesis by detecting and eliminating these bulges. First, *MSH2*-*MSH3* or *MSH2*-*MSH6* complexes, called MutSα or MutSβ, detect the error and recruit the *MLH1*-*PMS1*, *MLH1*-*PMS2* or *MLH1*-*MLH3* (MutLα/β/γ complexes respectively) to bind to the DNA and bring DNA exonuclease with *PCNA* to the mutation site (**Figure 1B**). The mismatch is then excised and repaired following by DNA resynthesis and re-ligation (33, 37). Since MSI-H cancers are defective in one of the MMR factors, mismatches in malignant cells remain unrepaired and accumulate indel mutations at high rates (18). Depending on where the microsatellite (MS) loci is located in the human genome, the effect of indel mutations differ: if it occurs within a non-coding segment of the genome, the indel mutation may have limited-to-no effect on overall gene expression or function; if it happens in a regulatory or splicing-required segment, the indel might affect linked gene expression; if it occurs within a protein-coding region the indel may result in expression of a truncated protein with novel peptide extension at the C-terminus (38). These cancer-specific peptide extensions called frameshifts can be exploited clinically in a variety of immunotherapy strategies which will be reviewed later.

Multiple biomarker strategies have been developed to detect either Lynch syndrome in the germline or assess penetration of MSI-H phenotype in tumors. Amsterdam II criteria and the Bethesda criteria are two clinically approved methods to diagnose Lynch syndrome in patients, and include a range of molecular tests with familial analysis of disease allele penetration (39). Several protocols exist to characterize MSI-H tumors: immunohistochemistry assay to quantify loss of MMR proteins such as *MLH1*, *MSH2*, *PMS2* and *MSH6*, genomic PCR tests to measure MS length variability at the various MS loci and quantification of somatic MS variation from WES/WGS data acquired from matched tumor/normal DNA samples using next generation sequencing and computational tools to calculate standardized MSI-H metrics like the “MSI score” (40–45). The strategies vary widely in terms of recall, sensitivity and specificity; thus, combining multiple protocols may increase the precision of the overall diagnosis (39, 46–48).

## MSI-H Cancer and Clinical Management

The standard of care for MSI-H cancer patients has changed from primarily chemotherapy to include immune checkpoint inhibitors. Approved for pMMR colorectal tumors, standard of care 5-fluorouracil (5-FU)-based regiments turned out to be ineffective in dMMR cases due to the lack of enzymes recognizing DNA damage inflicted by the drug (45, 49). Moreover, dMMR is turned out to be a negative prognostic biomarker for the objective response to 5-FU therapy and overall survival (49). Despite a setback in targeted therapy modalities for dMMR cancers, synthetic lethal interactions hold promise to improve the outcomes. dMMR tumors represent a good example of a system, where simultaneous co-inhibition of two factors may lead to cancer cell apoptosis and death. It has been found, that inhibition of Werner helicase is synthetically lethal with inactive *MSH2* or *PMS1* – the causative mutations of dMMR cancers. Mechanistically, the helicase is critical to unwind aberrantly replicated DNA and to maintain genome integrity of MSI-H tumors (50, 51). Pharmacological targeting of Werner helicase in dMMR cases still awaits its clinical application.

High tumor mutation burden (TMB) and comparatively strong immune cell infiltration of dMMR tumors sparked interest in applying checkpoint inhibitor (CPI) and other immunotherapy approaches (38, 52–54). Many studies confirmed a strong correlation between objective response rates to CPI and TMB in multiple cancers (55–58). A recent stage II multicohort clinical trial KEYNOTE-158 showed that TMB score is predictive for overall and progression free survival in the adjuvant CPI setting (59). Similarly, a range of clinical studies led by Dr. Diaz Jr. confirmed the strong association between responses to CPI and dMMR tumor status in a tissue-agnostic fashion (60, 61). Further investigation suggested dMMR status or MSI-H score to being predictive to CPI responses in solid tumors (62–64). These results led to the fast-track approval of dMMR as a biomarker for CPI by FDA and initiation of prospective stage II/III clinical trials, e.g., NCT02563002 or NCT04008030, to evaluate dMMR as a predictive biomarker. An interesting observation has arisen suggesting dMMR might be a confounding parameter for TMB biomarker in colorectal cancer patients treated with CPI: dMMR patients, which are often TMB-high due to intrinsic mechanisms of efficient somatic mutagenesis, clinically do better than “TMB-high but pMMR” patients (65). This clinical observation suggests the importance of the biological mechanism generating high somatic mutation load in tumor responsiveness to CPI. For instance, several hypotheses have been suggested to explain high CPI response rates in dMMR mechanistically. One of them is the “frameshift neoantigen” paradigm. Neoantigens are cancer-specific peptides that are usually derived from somatic mutations and presented on MHC-I or MHC-II complexes (66). dMMR tumors are enriched in frameshifts and neoantigens derived from these peptide extensions might confer tumor immunogenicity (67–70). Theoretically, one frameshift mutation may encode a peptide yielding multiple neoantigens with broad MHC-I/II specificities, thereby increasing the probability of (a) epitope presentation, (b) cancer antigen sampling by dendritic cells, (c) T cell recognition and (d) T cell-mediated tumor killing. We and others have shown exceptional immune responses against these frameshift peptides expressed in dMMR tumors, thus supporting the “frameshift neoantigen” paradigm (71–73). Another hypothesis is built around innate immune signaling driven by DNA instability. The aberrant DNA fragments are spilled over from the nucleus during DNA replication and recognized by *cGAS*-*STING* as “non-self” inducing Type I interferon and inflammatory NFkB responses (74, 75). In agreement with this hypothesis, recent study by Mowat et al. showed that *cGAS-STING* driven type I interferon signaling is required for *CXCL10*/*CCL5*-dependent T cell recruitment to dMMR tumor site (76). Both “frameshift-neoantigen” and “DNA instability” models are instructive and complementary in explaining the origins of the dMMR tumor immunogenicity (**Figure 1C**).

## Lynch Syndrome/MSI-H Cancer and “Off-The-Shelf” Vaccines

dMMR immunotherapies also include the implementation of cancer vaccine strategies in therapeutic and prospective settings (77). Several reasons suggest that such strategies might become successful. First, many dMMR associated somatic mutations are non-random across the genome. Known tumor drivers such as mutations in *APC*, *TP53* or *BRAF* genes are clonal, positively selected by the developing tumor and overrepresented within dMMR patient samples. Similarly, certain frameshift mutations are found to be under positive selection despite encoding immunogenic neoantigens (78, 79). From a tumor evolution perspective, such “cost” of an immunogenic frameshift can be accepted only if loss-of-function mutation confers a tumor growth advantage. Adaptive resistance against emerged neoantigens can be acquired later through general immune suppression mechanisms like *PDL-1* upregulation, infiltration of the TME by myeloid-derived suppressor cells or T regulatory cells, *TGF-β* expression or other genomically encoded immune-escape mechanisms (80). However, the positively selected frameshifts can be harnessed clinically as shared cancer vaccines (81, 82). Several targets including *RNF43^fs^*, *TGFBR2^fs^*, *ASTE1^fs^*, *AIM2^fs^*, have been extensively validated in numerous immunological assays: priming and boosting naïve T cell populations in healthy donors, confirming cytotoxic capacity of CD8+ T cell responses in tumor killing assays and detecting frameshift-specific memory responses within blood and tumor T cell compartments in dMMR cancer patients and Lynch syndrome populations (71, 83–85). The latter is particularly interesting because it suggests that immunological responses against frameshifts are observed in the absence of detectable cancer. In line with these results, a study lead by Dr. Kloor has documented dMMR mutations in crypts and polyps of Lynch syndrome patients which are not cancerous (86). These findings confirm that cancer development and loss of the mismatch repair system are two independent genomic events decoupled in time and may follow each other in any order (32). It also indicates that MS instability can happen early and progress without being recognized by the host immune system, likely because the majority of MS loci – targets of MS instability – are non-coding and spread over the human genome in a random fashion. Alternatively, instability of a protein coding MS locus does not necessarily lead to tumor transformation and might reflect a frequent, hotspot somatic passenger mutation such as those occurring in a healthy dividing cells (87, 88). It might take several cellular divisions and multiple MS instability events to develop a genetic background with an invasive tumor phenotype. Although, complete removal of dMMR cell populations might not be achieved due to the immune “invisibility” of such MS unstable clones, prospective vaccination against frequently observed and/or early detected frameshifts in dMMR cancer or dMMR Lynch syndrome patients is likely to increase the efficiency of immune surveillance of pro-tumorigenic cell populations and potentially delay tumor progression in the at-risk populations. Multiple vaccine formulations including different combinations of shared dMMR-associated frameshifts are currently undergoing safety and immunogenicity tests in clinical trials involving dMMR cancer and Lynch syndrome patients with established tumors (**Table 1**) (89, 90). It will be exciting to evaluate the clinical outcomes and cancer protection of Lynch syndrome patients in the prospective vaccine setting, similarly to the recently published antigen-agnostic immunomodulatory strategy involving naproxen – inhibitor of prostaglandin signaling (54). Recently published a proof-of-concept study confirmed therapeutic efficiency of a shared frameshift vaccine to delay tumor progression in mouse models of Lynch syndrome (91).

**Table 1:** List of registered clinical trials of cancer vaccines and/or CPI in dMMR/Lynch syndrome patients.

CPI and other immunotherapy clinical trials in dMMR cancers			
Study ID	Title	Status	Locations
NCT04612309	Retrospective Study on the Use of Immunotherapy in Patients With MSI-H Metastatic Colorectal Cancer	Recruiting	Italy
NCT04795661	Immunotherapy in MSI/dMMR Tumors in Perioperative Setting.	Not yet recruiting	France
NCT03827044	Avelumab Plus 5-FU Based Chemotherapy as Adjuvant Treatment for Stage 3 MSI-High or POLE Mutant Colon Cancer	Active, not recruiting	UK
NCT03206073	A Phase I/II Study of Pexa-Vec Oncolytic Virus in Combination With Immune Checkpoint Inhibition in Refractory Colorectal Cancer	Active, not recruiting	USA
NCT03150706	Avelumab for MSI-H or POLE Mutated Metastatic Colorectal Cancer	Active, not recruiting	South Korea
NCT03435107	Durvalumab for MSI-H or POLE Mutated Metastatic Colorectal Cancer	Active, not recruiting	South Korea
NCT04019964	Nivolumab in Biochemically Recurrent dMMR Prostate Cancer	Recruiting	USA
NCT02052908	Naproxen in Preventing DNA Mismatch Repair Deficient Colorectal Cancer in Patients with Lynch Syndrome	Completed	USA
**Clinical trials involving off-the-shelf cancer vaccines**			
NCT04799431	Neoantigen-Targeted Vaccine Combined With Anti-PD-1 Antibody for Patients With Stage IV MMR-p Colon and Pancreatic Ductal Cancer	Not yet recruiting	USA
NCT04117087	Pooled Mutant KRAS-Targeted Long Peptide Vaccine Combined With Nivolumab and Ipilimumab for Patients With Resected MMR-p Colorectal and Pancreatic Cancer	Recruiting	USA
NCT01885702	Dendritic Cell Vaccination in Patients With Lynch Syndrome or Colorectal Cancer With MSI	Active, not recruiting	Netherlands
NCT03152565	Avelumab Plus Autologous Dendritic Cell Vaccine in Pre-treated Metastatic Colorectal Cancer Patients	Completed	Spain
NCT04041310	Nous-209 Genetic Vaccine for the Treatment of Microsatellite Unstable Solid Tumors	Recruiting	USA
NCT01461148	Vaccination Against MSI Colorectal Cancer	Completed	Germany

Several considerations have to be taken during the development and application of a shared dMMR vaccine. General factors, including the platform selection (DNA, RNA, peptide), adjuvant, routes of administration and etc. – have been extensively reviewed elsewhere (92), but here we will discuss tumor intrinsic and potential acquired resistance mechanisms (93). As it has been mentioned above, dMMR tumors have enormous potential to develop somatic mutations through loss of DNA replication fidelity. dMMR tumors can be perceived as a “mutator” machine: cell population with an intrinsic mechanism to sample many different genotypes in a very rapid manner. Administration of external pressure such as through the vaccine-mediated expansion of tumor-specific T cell clones, may promote the development of tumor resistance against the host immune system. Several studies have reported up to 30% frequency of loss-of-function mutations in β-2-microglobulin (*B2M*) – a gene required for MHC-I antigen presentation and processing; up to 70% frequency of mutations in *TGFBR2* – cytokine receptor, rendering tumors non-responsive to *TGF-β* mediated suppression; up to 80% cumulative frequency of mutations in other genes related to innate and adaptive immune signaling pathways, namely *IFN-γ* response (*JAK1, JAK2*) and inflammasome activation (*CASP5*, *AIM2*). Other genomic mechanisms include loss of heterozygosity in *HLA-I* loci which drives tumor escape from CD8+ T cell cytotoxicity (94). Additionally, nonsense-mediated decay of the frameshifted messenger RNA can decrease immunogenicity against frameshift-derived neoantigens due to altered stability of the mutated RNA (95, 96). Non-genetic mechanisms of acquired resistance also can be found in the TME of the dMMR tumors, including: increased *Wnt*/β-catenin signaling in tumor associated fibroblasts; increased infiltration of *Foxp3+* T regs; upregulated expression of immune checkpoints *PDL-1* and *CTLA-4*; as well as *CD47* “don’t eat me” signals for macrophages and dendritic cells (93, 97, 98). Interestingly, a retrospective analysis of *B2M* expression and mutation status in colorectal dMMR cancer patients showed favorable clinical outcomes in patient cohorts despite *B2M* loss-of-function mutations, counterintuitive to the mechanisms of MHC-I dependance of immune-mediated tumor rejection (99, 100). A recent study by Germano et al. addressed this question and found CD4+ T cells being responsible for tumor rejection and the development of strong immune responses in *B2M*-null dMMR tumors (101). These and many other disparities between assumed inhibitory mechanisms and clinical outcomes in patients will inform many other mechanisms of therapy response and resistance which might exist in the dMMR cancer setting.

## Conclusions and Perspectives

Understanding the trajectories of dMMR tumor genome evolution at the single cell level with and without applied immune pressure will help to describe the landscape of acquired and intrinsic tumor resistance. Knowledge of these mechanisms might inform additional interventions important to include in the shared vaccine formulations such as MHC-II epitopes and/or NK cell engagers or myeloid cell modulation (38). CPI clinical trials conducted in dMMR patients can provide useful insights to address these questions. Discussed previously disparities between observed genomic alterations and immunotherapy clinical outcomes may inform novel mechanisms of immune resistance and response in dMMR tumors. Exploratory genetic and expression analysis of non-responder dMMR patient tumor samples from large-scale phase III CPI clinical trials will be highly informative to address these questions (60). Similarly, large scale sequencing and imaging data mining will be crucial to understand the mechanisms of dMMR tumor and immune cell dynamics. The majority of the detected frameshifts and MS loci indels are subclonal with relatively stable chromosomal copy number. If one specific mutation provides an immune resistance and doing so – growth advantage – why does not it become clonal during tumor evolution? A potential explanation is the uneven spatial tumor clone distribution and cooperativity between different tumor clones which might provide tumor benefit (102). Indeed, one can imagine the tumor surface lined up by clones governing immune resistance protecting other clones growing in the tumor core from infiltrating immune cells. In this “mutual dependency” scenario subclonal protective mutation will be sufficient to gain tumor growth advantage as a whole tumor cell community. Thus, spatially-resolved genomic studies combined with single-cell studies will be extremely informative to gain insight on spatial biomarkers associated with resistance and response to CPI and improve cancer vaccines designs by informing the inclusion of as many frameshifts derived from different tumor clones as possible (103).

In conclusion, we highlight several questions which remain important to address regarding treatment and prevention of MSI tumor in the near future. How many novel MS indels appear per each genome replication in dMMR lesions and/or dMMR Lynch syndrome crypts? What is the probability of acquiring a frameshift expressed at the protein level? Can sequence-based motifs predict the earliest frameshift to appear during dMMR development? Computational modelling leveraging whole genome MSI-H samples will be informative to answer these questions. Which frameshifts generate the most frequent immune responses *in vitro* and in dMMR cancer/Lynch syndrome patients? Which frameshift combination confers the best protective and cytotoxic potential in different cellular models of dMMR cancer progression? Extensive immunological studies will be very informative to address these points. Finally, characterizing and quantifying tumor intrinsic and acquired mechanisms of resistance from either clinical CPI trials or tumor model studies will be important to find alternative ways of improving therapeutic responses in patients’ populations. Overall, immunotherapeutic development to treat or protect against dMMR tumorigenesis experiences a new spiral of fruitful and exciting research.

## Author Contributions

VR wrote the manuscript. VR, CC, BG, AL, RS, and NB revised the manuscript. All authors contributed to the article and approved the submitted version.

## Funding

Support is provided by seed fund from Tisch Cancer Institute at Mount Sinai Hospital.

## Conflict of Interest

The authors declare that the research was conducted in the absence of any commercial or financial relationships that could be construed as a potential conflict of interest.

## Publisher’s Note

All claims expressed in this article are solely those of the authors and do not necessarily represent those of their affiliated organizations, or those of the publisher, the editors and the reviewers. Any product that may be evaluated in this article, or claim that may be made by its manufacturer, is not guaranteed or endorsed by the publisher.
